# A few north Appalachian populations are the source of European black locust

**DOI:** 10.1002/ece3.4776

**Published:** 2019-02-16

**Authors:** Xavier Paul Bouteiller, Cindy Frédérique Verdu, Emmi Aikio, Paul Bloese, Kasso Dainou, Adline Delcamp, Olivier De Thier, Erwan Guichoux, Coralie Mengal, Arnaud Monty, Marion Pucheu, Marcela van Loo, Annabel Josée Porté, Ludivine Lassois, Stéphanie Mariette

**Affiliations:** ^1^ BIOGECO, INRA Univ. Bordeaux Cestas France; ^2^ Biodiversity and Landscape Unit, Gembloux Agro‐Bio Tech University of Liège Gembloux Belgium; ^3^ Department of Genetics and Physiology University of Oulu Oulu Finland; ^4^ Department of Forestry Michigan State University East Lansing Michigan; ^5^ Forest Management Unit, Gembloux Agro‐Bio Tech University of Liège Gembloux Belgium; ^6^ Department of Botany and Biodiversity Research University of Vienna Vienna Austria

**Keywords:** biological invasion, bottleneck, introduction history, population genetics, *Robinia pseudoacacia*, single‐nucleotide polymorphism

## Abstract

The role of evolution in biological invasion studies is often overlooked. In order to evaluate the evolutionary mechanisms behind invasiveness, it is crucial to identify the source populations of the introduction. Studies in population genetics were carried out on *Robinia pseudoacacia *L., a North American tree which is now one of the worst invasive tree species in Europe. We realized large‐scale sampling in both the invasive and native ranges: 63 populations were sampled and 818 individuals were genotyped using 113 SNPs. We identified clonal genotypes in each population and analyzed between and within range population structure, and then, we compared genetic diversity between ranges, enlarging the number of SNPs to mitigate the ascertainment bias. First, we demonstrated that European black locust was introduced from just a limited number of populations located in the Appalachian Mountains, which is in agreement with the historical documents briefly reviewed in this study. Within America, population structure reflected the effects of long‐term processes, whereas in Europe it was largely impacted by human activities. Second, we showed that there is a genetic bottleneck between the ranges with a decrease in allelic richness and total number of alleles in Europe. Lastly, we found more clonality within European populations. Black locust became invasive in Europe despite being introduced from a reduced part of its native distribution. Our results suggest that human activity, such as breeding programs in Europe and the seed trade throughout the introduced range, had a major role in promoting invasion; therefore, the introduction of the missing American genetic cluster to Europe should be avoided.

## 1 INTRODUCTION

Since their first definition in Charles Elton's book (Elton, 1958), biological invasions have been increasingly studied over the last few decades. Compared to the ecological impacts of many invasive species and the management issues surrounding them, the role of evolution in biological invasions has long been overlooked (Colautti & Lau, 2015). In order to fill this knowledge gap, it is crucial to identify the source populations of the introduction for a better understanding of the evolutionary mechanisms behind invasiveness, such as the role of selection, local adaptation or admixture (Colautti & Lau, 2015; Dlugosch, Anderson, Braasch, Cang, & Gillette, 2015; Keller & Taylor, 2008). The practical applications of such studies are the identification of source risk and the prediction of the invasive potential of a population (Chown et al., 2015).

When a species is introduced to a new range, it is generally expected to experience a genetic bottleneck leading to a loss of genetic diversity (allelic richness or heterozygosity) (Dlugosch et al., 2015). For example, the invasive plant *Heracleum mantegazzianum* exhibited a lower diversity in the invasive range attesting a strong founder event (Henry et al., 2009). However, some studies (Dlugosch et al., 2015; Dlugosch & Parker, 2008) have emphasized that the loss of genetic diversity within the native and invasive ranges was generally weak (15%–20% on average); this can be explained by multiple introductions that have limited the loss of diversity, as in the case of *Phalaris arundinaceae* or *Prunus serotina *(Lavergne & Molofsky, 2007; Pairon et al., 2010). Genetic diversity is even likely to increase in the invasive range if population admixture is high (Dlugosch et al., 2015; Dlugosch & Parker, 2008), although a large increase is rare (Uller & Leimu, 2011); for example, the invasion of *Phalaris arundinacea *was shown to have been promoted by an increased genetic variation (Lavergne & Molofsky, 2007). Genomic admixture was likely to have favored the success of *Silene vulgaris* in its new American range (Keller, Fields, Berardi, & Taylor, 2014; Keller & Taylor, 2010).

Additionally to propagule pressure during introduction, the mating system can have a high impact on the diversity and genetic structure of populations. Clonal or self‐fertilizing species are likely to experience a greater loss of genetic diversity, whereas a bottleneck effect may be reduced for outcrossing species (Baker, 1967; Pappert, Hamrick, & Donovan, 2000). For example, the loss of genetic diversity between native and invasive ranges was greater for purely clonal populations of the invasive *Oxalis pes‐caprae *than for sexual ones (Ferrero et al., 2015).

Few studies have been carried out on the numerous invasive species of trees and shrubs, despite their great impact on ecosystems (Richardson & Rejmánek, 2011). Contrary to many herbaceous species, invasive trees (comprising 357 tree species, i.e., nearly 0.5% of all trees species; Richardson & Rejmánek, 2011) have often been voluntarily introduced to their new ranges for horticultural or forestry purposes (Richardson & Rejmánek, 2011), resulting in multiple repeated introductions which may have shaped the diversity of the trees in the introduced range (Hirsch, Richardson, & Le Roux, 2017). Furthermore, invasive trees are characterized by a longer generation time compared to invasive herbaceous species; this life‐history trait may influence differentiation rate between ranges. In addition, the fact that a few centuries and tree generations have passed since the first introduction presents a challenge to the study of evolutionary processes in invasive trees (Hirsch et al., 2017). Little research has been carried out on the population genetics of invasive trees in both their invasive and native ranges. To our knowledge, a few studies have evidenced mostly multiple introductions to the invasive range from the native range; for example, *Acacia saligna* (Thompson, Bellstedt, Richardson, Wilson, & Le Roux, 2015) and *Pinus tadea *(Zenni, Bailey, & Simberloff, 2014) have been widely sampled from the native range exhibiting a high level of admixture within the invasive range, and *Prunus serotina* (Pairon et al., 2010) has been introduced several times, but from a limited part of the native range.

Black locust *Robinia pseudoacacia* L. (Fabaceae) is native to North America and is considered as invasive on a world scale (eight regions among the 14 defined by Richardson & Rejmánek, 2011). In Europe, it is now recognized as one of the 100 worst invasive species (Basnou, 2009; DAISIE, 2006).

The native range of this species consists of two disjoint areas on both sides of the Mississippi watershed (Little, 1971); the largest area corresponding to the Appalachian Mountains and partially encompassing several current States (Pennsylvania, Maryland, West Virginia, Virginia, North Carolina, South Carolina, Georgia, Alabama, Tennessee, Kentucky, and Ohio) and the smallest area being located further west in the Ozark Mountains (Missouri, Arkansas and Oklahoma). In America, black locust was intensively displaced by settlers due to the undeniable interest in its wood as stated by Michaux in “*Histoire des arbres d'Amériques septentrionales tome III*” (1813), or by Cobbett in his book “The Woodlands” (1825). Such displacement has sometimes led to the misinterpretation of its native distribution; for example, in the Gardeners Dictionary (1756–1759), Miller wrongly stated that black locust was native to Massachusetts (Michener, 1988). To date, this species has spread to every state in the contiguous USA and also to British Columbia, Québec, Newfoundland and Labrador in Canada (Schütt, 1994). It was introduced to Europe during the early 17th century and it is now present in all European countries; however, when and how the first introduction occurred is not precisely known and is still shrouded in mystery. It is widely written that Jean Robin (1550–1629), botanist of King Henri the Fourth in 1601, was responsible for the first European introduction. A century later, Carl Von Linné gave the black locust its current name, *Robinia*, in recognition of the work carried out by Jean Robin and son (Vespasien) in acclimating it to Europe. Actually, 1601 seems a very unlikely date since black locust is absent (Wein 1930 cited by Cierjacks et al., 2013) from the lists edited by Jean and Vespasien Robin in 1601 (“*catalogue de son jardin*”) and in 1620 (“*histoire des plantes nouvellement trouvées en l'isle de Virginie*”). To our knowledge, the first citations of the species appeared in England in the John Tradescant list in “*plantarum in horto Iohannem Tradescanti*” (1634) (cited in “Early British botanists and their gardens” by Gunther, 1922) under the name of *Locusta virginiana arbor*, and quasi‐simultaneously in France in the book *Plantarum canadesium historia* (1635) by Vespasien Robin's friend, Jacques Philippe Cornuti, under the name of *Acacia americana robini*. Moreover, Tradescant's son travelled to Virginia before 1640 (Hamel, 1854) and as it has been documented that he brought several plants back with him, it is likely that he was the one who first introduced black locust seeds to Europe from Virginia. It has been established that Tradescant the elder corresponded and exchanged seeds with the Robins from 1601 (Gunther, 1922); thus he probably sent some seeds to Vespasien Robin in France, who would have sown them and cultivated the trees (such as the one planted in the King's garden in Paris in 1634 (“*jardin des plantes*”; in Biographie Universelle, 1824)).

In Europe, the first introduction appears to have been followed by a period of interest in its ornamental aspect; however, it subsequently fell into disuse in the early 18th century, as explained in a dictionary from 1722 about the black locust, which was quoted by Nicolas François de Neufchateau in his book “*lettre sur le robinier*” (1807). In the middle of the 18th century, American explorers returned to Europe and promoted the use of black locust in forestry: For instance, Michaux (1813) described the abundance of this tree in the Allegheny mountains throughout Pennsylvania and West Virginia and indicated that after the end of the 18th century, the tree was appreciated more for the excellent qualities of its wood than for the beauty of its foliage and flowers. At the same time, the English politician William Cobbett, who emigrated to America in the late 18th century, emphasized all the qualities of this tree and promoted its plantation in Europe: “I sold the plants; and, since that time, I have sold altogether more than a million of them,” adding that “My seed has always come from the neighborhood of Harrisburgh in Pennsylvania” (Cobbett, 1825).

From this information, we can conclude that the European dissemination of the black locust seems to have experienced a lag phase between the tree species’ first introduction to Europe—possibly from Virginia during the early 17th century—and its rediscovery in the middle of the 18th century, leading to a new wave of introductions of the species, which probably came from Pennsylvania and the Virginias. More recently, black locust breeding programs have been carried out in central Europe since the beginning of the 20th century (Keresztesi, 1983; Liesebach, Yang, & Schneck, 2004; Straker, Quinn, Voigt, Lee, & Kling, 2015). Currently, Hungary is the European leader in the production of black locust seedlings, and their selected provenances for wood production are now widely distributed in Europe for new forest plantations (Keresztesi, 1983; Liesebach et al., 2004; Straker et al., 2015). In Europe, the black locust is now recognized as one of the 100 worst invasive species (Basnou, 2009; DAISIE, 2006) and it is considered as an invasive tree on a world scale (eight regions out of the fourteen defined by Richardson & Rejmánek, 2011).

Although knowledge about the black locust's genetic diversity is key to developing further ecological or evolutionary studies (Lawson Handley et al., 2011), little information exists about its genetic diversity and structure in introduced ranges, nor regarding its origin and differentiation from the population sources in North America. The only studies we know of in Europe compared four American populations with sixteen German and Hungarian populations (Liesebach & Schneck, 2012; Liesebach et al., 2004), but although the results suggested a high genetic differentiation among American populations, they were mostly inconclusive. Modern molecular and statistical tools used in population genetics have proved to be useful for finding the geographic origins of invasive species, complementing or providing a solution to the lack of available historical knowledge (Besnard et al., 2014; Chown et al., 2015; Cristescu, 2015; Hoos, Whitman Miller, Ruiz, Vrijenhoek, & Geller, 2010). Using SNP markers developed for the black locust (Verdu et al., 2016), we investigated its introduction history and genetic diversity in its native range and European invasive range, in particular by answering the following questions: (a) Can we identify the native population sources of European black locust? (b) What is the genetic differentiation within and between ranges? (c) Can we detect a founder event associated with a loss of genetic diversity?

## 2 MATERIAL AND METHODS

### 2.1 Sampling

Sixty‐three populations of black locust were sampled in both the native range (29 populations) and the European invasive range (34 populations). Sampling was conducted between spring 2014 and fall 2016 (Table 1 and Supporting Information Appendix S1) by different collaborators using the same protocol: Between 10 and 30 trees were sampled in each population. Samples were collected either in common gardens or in natural populations. A total of 818 individuals were sampled: 402 from Europe and 416 from North America.

**Table 1:** General genetic information regarding the sampled populations

Number	Range	Country/State	Pop	*X* Long	*Y* Lat	*N*	*G*	*R*	*F*_IS_ Mean	*F*_IS_ LCI95	*F*_IS_ HCI95	Ho	Hs
1	EU	France	Barthelasse Avignon	4.818	43.965	19	18	0.944	**0.112**	0.048	0.179	0.232	0.262
2	EU	Czech Republic	Brno	16.518	49.042	11	5	0.400	−0.026	−0.131	0.084	0.240	0.234
3	EU	Hungary	Budapest	19.107	47.663	20	13	0.632	**0.115**	0.047	0.184	0.244	0.276
4	EU	Romania	Carei	22.449	47.661	11	11	1.000	**0.096**	0.026	0.165	0.259	0.286
5	EU	Belgium	Corphalie	5.259	50.539	10	10	1.000	**0.120**	0.042	0.195	0.251	0.285
6	EU	Poland	Drewnica	21.251	52.253	10	10	1.000	0.060	−0.015	0.138	0.231	0.246
7	EU	Spain	Gafos Galicia	−8.617	42.383	12	5	0.364	0.056	−0.047	0.166	0.269	0.285
8	EU	Bulgaria	Gorna Oryahovitsa	25.694	43.119	12	12	1.000	**0.100**	0.026	0.176	0.246	0.273
9	EU	Netherland	Kelpen‐Oler	5.825	51.205	12	12	1.000	**0.119**	0.039	0.197	0.245	0.278
10	EU	Germany	Klein	9.089	49.991	11	11	1.000	0.010	−0.099	0.124	0.240	0.243
11	EU	France	La Flotte	−0.305	44.385	6	3	0.400	0.062	−0.087	0.205	0.240	0.259
12	EU	France	La Gouaneyre	−0.273	44.376	6	5	0.800	**0.152**	0.020	0.281	0.213	0.251
13	EU	England	London Streat Ham	0.143	51.433	10	7	0.667	**0.144**	0.023	0.265	0.239	0.279
14	EU	England	London Wandworth	0.163	51.446	10	8	0.778	**0.085**	0.002	0.166	0.240	0.262
15	EU	Macedonia	Macedonia	21.571	41.507	12	12	1.000	**0.086**	0.017	0.157	0.242	0.265
16	EU	Germany	Meppen	7.377	52.704	12	8	0.636	−0.097	−0.211	0.026	0.257	0.235
17	EU	Spain	Montseny	2.512	41.831	12	12	1.000	0.068	−0.009	0.147	0.230	0.246
18	EU	Germany	Munchenberg	14.046	52.559	12	4	0.273	**−0.180**	−0.319	−0.030	0.223	0.190
19	EU	Bulgaria	Novi Pazar‐Kulevcha	27.195	43.345	12	12	1.000	**0.091**	0.024	0.161	0.242	0.267
20	EU	Hungary	Nyirsegi	19.041	47.581	12	12	1.000	**0.087**	0.004	0.173	0.242	0.265
21	EU	Germany	Obermeidenrich	6.816	51.476	12	12	1.000	**0.066**	0.000	0.134	0.254	0.272
22	EU	Poland	Pinczow	20.702	50.265	10	10	1.000	**0.154**	0.067	0.249	0.212	0.251
23	EU	Poland	Poznan	16.808	52.311	12	12	1.000	**0.119**	0.037	0.203	0.230	0.261
24	EU	Germany	Priesterweg Naturpark	13.358	52.461	12	12	1.000	**0.105**	0.035	0.177	0.249	0.278
25	EU	Hungary	Pusztavacs	19.506	47.172	10	10	1.000	0.059	−0.021	0.147	0.256	0.273
26	EU	France	Remy	2.675	49.460	12	8	0.636	**0.104**	0.014	0.193	0.227	0.254
27	EU	Greece	Rhodos	27.944	36.287	12	9	0.727	**0.104**	0.014	0.197	0.227	0.254
28	EU	Slovakia	Slovakia	19.867	*48.720*	12	12	1.000	**0.161**	0.075	0.251	0.218	0.260
29	EU	Poland	Szczecin	14.548	53.337	12	12	1.000	**0.076**	0.003	0.151	0.242	0.262
30	EU	Turkey	Turkey	32.904	*40.159*	11	11	1.000	0.081	−0.005	0.172	0.224	0.245
31	EU	Netherland	Uden	5.618	51.685	11	5	0.400	**0.133**	0.015	0.247	0.242	0.280
32	EU	Spain	Valencia	−0.784	39.397	19	6	0.278	0.066	−0.048	0.179	0.247	0.264
33	EU	Spain	Vitoria	−1.942	43.216	13	13	1.000	**0.132**	0.054	0.210	0.237	0.273
34	EU	Austria	Wien	16.473	48.252	12	12	1.000	0.069	−0.013	0.155	0.249	0.268
35	US	PA—Pennsylvania	ALTOONA	−78.383	40.489	11	10	0.900	**0.115**	0.036	0.192	0.249	0.281
36	US	VA—Virginia	Barbours Creek	−80.110	37.580	21	20	0.950	**0.126**	0.064	0.192	0.215	0.246
37	US	NC— North Carolina	Blue Ridge	−82.672	35.457	22	21	0.952	**0.118**	0.060	0.176	0.210	0.238
38	US	WV—West Virginia	CAMP CREEK	−81.103	37.488	12	12	1.000	0.073	−0.008	0.154	0.237	0.255
39	US	TN—Tennessee	CHATTANOOGA	−85.783	35.120	12	11	0.909	0.065	−0.020	0.151	0.210	0.224
40	US	KY—Kentucky	DANIEL BOONE NF	−83.645	37.751	12	12	1.000	**0.147**	0.067	0.230	0.222	0.260
41	US	KY—Kentucky	Eriline	−83.540	37.040	22	22	1.000	**0.082**	0.027	0.138	0.222	0.241
42	US	AR—Arkansas	FAYETTEVILLE	−94.205	36.071	10	9	0.889	**0.186**	0.095	0.277	0.202	0.248
43	US	WV—West Virginia	FORT MILL RIDGE	−78.797	39.327	12	12	1.000	0.056	−0.012	0.124	0.236	0.250
44	US	AR—Arkansas	FORT SMITH	−94.290	35.343	12	10	0.818	**0.117**	0.027	0.215	0.188	0.213
45	US	OH—Ohio	Ironton	−82.460	38.800	22	17	0.762	**0.105**	0.033	0.181	0.231	0.258
46	US	WV—West Virginia	LEWISBURG	−80.381	37.783	7	7	1.000	0.037	−0.061	0.137	0.239	0.249
47	US	NC—North Carolina	Locust Cove	−83.710	35.360	22	18	0.810	**0.115**	0.056	0.179	0.213	0.241
48	US	KY—Kentucky	Morehead	−83.466	38.091	22	19	0.857	**0.135**	0.064	0.209	0.196	0.226
49	US	AR—Arkansas	OUACHITA	−93.837	34.449	12	9	0.727	**0.097**	0.015	0.178	0.230	0.254
50	US	WV—West Virginia	Perry	−78.660	39.000	22	22	1.000	**0.097**	0.026	0.168	0.229	0.253
51	US	AR—Arkansas	Pleasant Hill	−93.460	35.590	4	3	0.667	−0.058	−0.200	0.099	0.245	0.234
52	US	NC—North Carolina	SHOOTING CREEK	−83.628	35.055	11	11	1.000	**0.111**	0.039	0.186	0.209	0.235
53	US	WV—West Virginia	Slatyfork	−80.000	38.180	17	17	1.000	**0.108**	0.040	0.175	0.215	0.242
54	US	VA—Virginia	Stokesville	−79.300	38.280	22	22	1.000	**0.131**	0.060	0.206	0.204	0.235
55	US	MD—Maryland	US GRP 1	−78.750	39.650	15	15	1.000	**0.095**	0.030	0.159	0.224	0.247
56	US	WV—West Virginia	US GRP 2	−81.100	39.067	11	11	1.000	**0.133**	0.052	0.216	0.214	0.247
57	US	VA—Virginia	US GRP 3	−79.933	37.267	6	6	1.000	**0.123**	0.018	0.228	0.247	0.281
58	US	KY—Kentucky	US GRP 4	−84.500	38.033	6	6	1.000	0.095	−0.001	0.196	0.206	0.228
59	US	KY—Kentucky	US GRP 5	−84.533	38.650	8	8	1.000	**0.175**	0.084	0.268	0.200	0.242
60	US	KY—Kentucky	US GRP 6	−83.683	36.750	7	7	1.000	**0.157**	0.047	0.260	0.215	0.255
61	US	AR—Arkansas	Victor	−93.050	35.650	22	17	0.762	**0.151**	0.085	0.224	0.193	0.227
62	US	OH—Ohio	WAYNE NF	−82.594	38.658	12	12	1.000	**0.132**	0.049	0.216	0.222	0.256
63	US	VA—Virginia	Whiteop	−81.656	36.769	22	20	0.905	**0.114**	0.049	0.183	0.217	0.245

Note

The range corresponds either to Europe (EU) or the USA (US) and either the country or the state is indicated. *X* Long and *Y* Lat (longitude and latitude, respectively) corresponded to the GPS coordinates of the sampled population provided in the WGS84 geographic projection. *N* is the number of individuals genotyped per population. *G* is the number of unique genotypes in each population. *R* is the index of clonal diversity, as defined in the material and methods section. *F*_IS_ mean, *F*_IS_ LC95 and *F*_IS_ HC95 indicate, respectively, mean *F*_IS_ value and the 95% confidence interval computed using the hierfstat R package for each population. The *F*_IS_ values in bold indicate that the 95% confidence interval, calculated using 1,000 bootstrap replicates, does not include zero. Ho is the observed heterozygosity and Hs the expected heterozygosity. Genetic diversity values were calculated using the initial dataset after clone removal (i.e., 113 SNPs and 720 individuals).

Black locust propagates through sexual and asexual reproduction. In common gardens, since trees were grown from seeds of known origin, there was no risk of collecting clones. However, in natural populations, a minimal distance of 25 m was kept between two sampled trees in order to minimize the risk of collecting clones.

Either leaves, cambium, buds, or seeds were harvested depending on the season. For leaf sampling, a few leaflets on a green healthy leaf were collected using a manual tree pruner. For cambium sampling, external bark was removed from the trunk with a knife, then five rings of wood were collected using a 1‐cm‐diameter punch. In the field, samples were put into referenced tea bags and then placed into plastic boxes containing silica gel in order to dry the samples. The silica gel was renewed after 24 hr and 48 hr and then until it no longer changed color. The plastic boxes were then stored at ambient temperature in closed cupboards.

In natural populations, GPS coordinates of either the population or each sampled tree were recorded using a portable GPS (GPSMAP62, Garmin, Olathe, KS, USA). On the campus of Michigan State University, the geographic origins of each mother tree were known and were used for the coordinates of the sampled trees, and the populations were defined by gathering trees from a close geographic location (see Supporting Information Appendix S1).

### 2.2 DNA extraction and genotyping

For each individual, either a 1 cm^2^ leaf sample was collected on a leaflet, cambium was manually extracted from one ring of wood or five buds were collected. The plant material was then crushed using an automated grinder (2010 Geno/Grinder, SPEX SamplePrep, Metuchen, NJ, USA). For four populations (Corphalie, Drewnica, Pinczow and Lewisburg), a few seeds from ten sampled mother trees were scarified and grown in the laboratory (Bouteiller, Porté, Mariette, & Monty, 2017). The first fresh leaf on each specimen was then used for genotyping. DNA was extracted and isolated from all populations using DNeasy 96 Plant Kit (Qiagen, Venlo, Netherlands) following the manufacturer's protocol. One negative control was set on each plate. DNA concentration was measured using an UV spectrophotometer NanoDrop 8000 (Thermo Fisher Scientific Inc., Wilmington, Delaware, USA) and confirmed using Quant‐iT™ dsDNA Assay Kit (Thermo Fisher Scientific Inc., Wilmington, Delaware, USA). Besides DNA concentration, 260/280 and 260/230 absorbance ratio provided information about DNA purity. DNA concentrations were standardized to 10 ng/µl before SNP genotyping.

SNPs have recently been developed on black locust (Verdu et al., 2016) using the double‐digest RAD approach. Nine samples (from six North American trees, two European trees, and one Iranian tree) were digested with *Eco*RI/*Mse*I and subsequent libraries were sequenced using Illumina technology. The resulting sequences were submitted to a bioinformatics pipeline and more than 300 SNPs were validated by carrying out individual genotyping using the Sequenom MassARRAY System (Agena Bioscience, San Diego, USA). It is assumed that the SNPs used in the present study were located within neutral regions since we did not use restriction enzymes targeting nonneutral restriction sites.

Two genotyping experiments were performed using these SNPs: (a) All collected samples (initial dataset) were genotyped using 113 SNPs, which were selected according to the procedure presented in Bouteiller et al. (2018); (b) after clone removal (see Section 3), 163 individuals were subsampled randomly within the populations in each range to maintain the sampling design between ranges (additional dataset: 69 individuals from the USA and 96 from Europe) and genotyped using a total of 251 SNPs. These additional SNPs were among the SNPs developed by Verdu et al. (2016), which had first been discarded as we had initially chosen to prioritize the more polymorphic SNPs. Four additional multiplexes of SNPs (138 SNPs in total) were thus designed using the MassArray Assay Editor 4.0.1.4 software (see Verdu et al., 2016 for more details regarding the procedure). SNP genotyping was performed using the Sequenom MassARRAY System (Agena Bioscience, San Diego, USA) at the Bordeaux Genome Transcriptome Facility (https://pgtb.cgfb.u-bordeaux.fr/en), and using the iPLEX Gold chemistry genotyping kit according to the manufacturer's instructions. SNP data were visualized and validated using ViClust, a R program that we implemented for Galaxy (https://usegalaxy.org/); the program was also made available as a standalone R script for Linux or Windows (Bouteiller et al., 2018). The whole dataset will be made available on the Open Science Framework repository after acceptation.

### 2.3 Data analysis

#### 2.3.1 Clone removal

For the analysis of genetic diversity and structure within and between ranges, we chose to identify and remove clones from the analysis using R version 3.3.1 (R Development Core Team, 2016). Within populations, only markers without missing values were kept, and a pairwise comparison of each genotyped individual was carried out in order to detect putative clones.

For each population, the index of clonal diversity R (Arnaud‐Haond, Duarte, Alberto, & Serrão, 2007) was calculated as:

(1) $$R=\frac{\left(G-1\right)}{\left(N-1\right)}$$

where *G* is the number of unique genotypes in the considered population and *N* the sample size of the population. This index varies from 0 in purely clonal populations to 1 when all the individuals corresponded to different genotypes.

As some populations were sampled from trees in common gardens or from laboratory‐grown seedlings which originated from seeds (Table 1), they were unlikely to contain clones; however, we checked that no clone was present in these populations and removed them before carrying out the subsequent analysis. The difference in clonality between ranges was determined using a Pearson *χ* squared test with Yate's continuity correction using R version 3.3.1 (R Development Core Team, 2016).

#### 2.3.2 Molecular genetic structure

After removing clones from the dataset, molecular genetic differentiation was explored both between ranges and among populations within ranges using two approaches.

First, the typology of all sampled individuals from both ranges was assessed using a principal component analysis (PCA), developed in the R adegenet library (Jombart, 2008; Jombart, Devillard, & Balloux, 2010).

Second, individual membership was analyzed using the Bayesian clustering approach, *Markov Chain Monte Carlo* (MCMC), developed in the STRUCTURE v2.3.4 software (Porras‐Hurtado et al., 2013; Pritchard, Stephens, & Donnelly, 2000), using an admixture model. Each model run assumed that the overall diversity was structured into *K* clusters and, according to the SNP data, each individual was assigned proportionally to each cluster; thus, for each individual membership we obtained coefficients with a 90% confidence interval for the associated *K* clusters by setting the ANCESTDIST parameter from 0 to 1.

Each run corresponded to a MCMC model with a burn‐in period of 500,000 iterations followed by 500,000 iterations, which was repeated 10 times (Gilbert et al., 2012). The analysis was first performed using the initial dataset after clone removal to determine the structure of populations from both ranges (*K* varying from 1 to 20), then it was performed for each range separately (*K* varying from 1 to 15). The most probable number of clusters was determined according to Evanno, Regnaut, & Goudet (2005) using the peak in the Δ*K* parameter calculated with the STRUCTURE HARVESTER software (Earl & vonHoldt, 2012). All runs were computed on the GenoToul bioinformatics cluster (http://bioinfo.genotoul.fr/) using the StrAuto script (Chhatre & Emerson, 2017) to produce STRUCTURE mainparams and extraparams and to automatize and parallelize the STRUCTURE analysis. Finally, the CLUMPAK software (Kopelman, Mayzel, Jakobsson, Rosenberg, & Mayrose, 2015) was used to synthetize STRUCTURE outputs and compute graphs of membership into the most likely *K* cluster number, in the form of line charts for individuals or pie charts for populations.

We determined if each individual was significantly assigned to one of the *K* clusters or if it was admixed by using individual membership coefficient confidence intervals for the *K* clusters. When the confidence intervals were not overlapping, an individual was considered as significantly assigned to the *K*th cluster for which it had the highest membership coefficient. Thus, for each population we were able to calculate the ratio of individuals assigned to each *K*th cluster or which were admixed. Finally, we assigned the population to the cluster for which the proportion of assigned individuals was the highest. In the case of equality, or if 100% of individuals were admixed, the population was declared as admixed.

To visualize within range genetic structure, we computed spatial interpolation using individual membership coefficients for the most likely *K* within each range. An inverse distance weighting (IDW) interpolation with a power value of 1 was carried out using the ArcGIS v10.2.2 geostatistical analyst tool (ESRI, 2011). The neighborhood was searched using a four sectors circle with a maximal value of 25 neighbors and a minimum of 0.

#### 2.3.3 Analysis of genetic differentiation and diversity

The genetic differentiation between populations (with values between 0 and 1, none‐full differentiation) was analyzed using *F*_ST_ indexes (Wright, 1931). Within and between ranges, *F*_ST _were calculated with the hierfstat v 0.04‐28 R package (Goudet, 2005) according to the Weir and Cockerham method (1984). 95% confidence intervals (CI) were estimated by performing 1,000 bootstraps over loci.

Two datasets were analyzed in order to compare genetic diversity between ranges: the initial dataset (818 individuals minus 98 clones = 720 genotyped individuals using 113 SNPs) and the additional dataset (163 genotyped individuals using 251 SNPs, see Section 2). The second dataset specifically aimed to test for a potential bias in allelic frequency due to SNP selection. First, allelic frequencies were evaluated by plotting the MAF (Minor Allelic Frequency) distributions per locus and per range. Using the R package Hierfstat v 0.04‐28 (Goudet, 2005) and Fstat software v2.9.3 (Goudet, 2013), diversity indices were calculated (a) between ranges, (b) among populations within ranges, and (c) within populations and were as follows: observed heterozygosity (Ho), which quantifies the proportion of heterozygous individuals; expected heterozygosity (He), also known as genetic diversity, that measures the expected proportion of heterozygous individuals under Hardy–Weinberg Equilibrium; allelic richness (AR), which corresponds to the number of alleles weighted by the number of individuals in the smallest population; inbreeding coefficient *F*_IS_ within each population that measures the proportional deviation of observed from expected heterozygosity within subpopulation; and the total number of alleles (TNA) per range, calculated by summing within each range the number of allele over all loci.

Differences in AR were determined by performing a nonparametric Wilcoxon paired test among loci between ranges. In order to evaluate differences in total number of alleles between ranges, a bootstrap over all loci and individuals was computed using 1,000 simulations and the differences were determined using a nonparametric Mann–Whitney test using R version 3.3.1 (R Development Core Team, 2016).

#### 2.3.4 Isolation by distance analysis

In natural populations, genetic similarity is expected to be high between spatially close populations and then to decrease among populations with geographic distance; this pattern is known as isolation by distance (IBD). IBD was tested within each range, using two approaches: (a) The genetic distances between populations were calculated as the ratio *F*_ST_/(1−*F*_ST_) using pairwise *F*_ST_ plotted against the logarithm of the pairwise geographic distances among populations (Rousset, 1997) and the correlation was tested using a Pearson coefficient test; and (b) A Mantel test between the matrices of pairwise geographic distances and pairwise genetic distances was performed using the R ade4 library, with 9,999 permutations of matrices. For both methods, pairwise geographic distances among populations were calculated with the GPS coordinates of each population using the R CalcDists function provided by Scott Chamberlain on GitHub (https://gist.github.com/sckott/931445). The matrix of pairwise genetic distances was estimated using the Cavalli‐Sforza and Edwards Chord distance with hierfstat v 0.04‐28 (Takezaki & Nei, 1996).

## 3 RESULTS

### 3.1 More asexual reproduction in European populations

Overall, a higher clonality was detected in the European populations compared to the American ones, with a significant range effect (*χ* ^2^ = 29.04, *df* = 1, *p* = 7.10 × 10^−8^). As expected, no clone was found within the common garden populations, nor in the populations obtained from seedlings germinated in the laboratory. When removing these populations from the analysis (thus leaving 280 European and 356 American individuals), 98 genotypes were found with a least one duplicated version (i.e., clones): 68 clones out of the 280 European samples and 30 clones out of the 356 American samples. Keeping only one sample per genotype resulted in a dataset that contained 720 genotypes out of the 818 sampled individuals after clone removal, which was distributed as 334 genotypes in 34 European populations and 386 genotypes in 29 American populations.

Among European populations (Table 1), the index of clonal diversity R ranged from 0.27 to 1 (mean = 0.82, *SD* = 0.26), with significant differences in clonality between populations (*χ* ^2^ = 88.33, *df* = 60, *p* = 0.01). The most clonal populations (*R* < 0.5) were Munchenberg Germany (*R* = 0.27), Valencia Spain (*R* = 0.28), Gafos Galicia Spain (*R* = 0.36), Brno Czech republic (*R* = 0.4), La Flotte France (*R* = 0.4), and Uden the Netherlands (*R* = 0.4). Among American populations, the index of clonal diversity *R* ranged from 0.67 to 1 (mean = 0.93, *SD* = 0.10) and there was no significant effect of population on the clonality (*χ* ^2^ = 76.77, *df* = 60, *p* = 0.07). The lowest *R* value was observed for Pleasant Hill, in Arkansas (*R* = 0.67).

The overall *F*_IS_ that was calculated among European populations of the “clonal dataset” (0.019, 95%CI: −0.018–0.062, estimated by bootstrapping over loci) was significantly lower than the overall *F*_IS_ calculated among American populations of the “clonal dataset” (0.11, 95%CI: 0.077–0.14, estimated by bootstrapping over loci). At the population level, negative *F*_IS_ values (Table 1) were observed in three European populations (Brno: −0.0256, Meppen: −0.0974, Munchenberg: −0.180) and in one American population (Pleasant Hill: −0.0582) but only Munchenberg was significant. All these populations exhibited high clonal reproduction (*R* ranging from 0.28 to 0.67). Significantly positive *F*_IS_ values were estimated among 22 of the 34 European populations (ranging from 0.066 to 0.161) and among 23 of the 29 American populations (ranging from 0.082 to 0.186).

### 3.2 Introduced populations are genetically close to a few northeastern native populations

Introduced European individuals of black locust are genetically close to only a few native individuals located in four populations from the northeastern part of its native range. Among the 29 sampled American populations, only four (Altoona, Eriline, US Grp 3, and Wayne National Forest) showed individuals with a high level of genetic relatedness to the European individuals. The structure and multivariate analyses gave congruent results.

**Figure 1:**
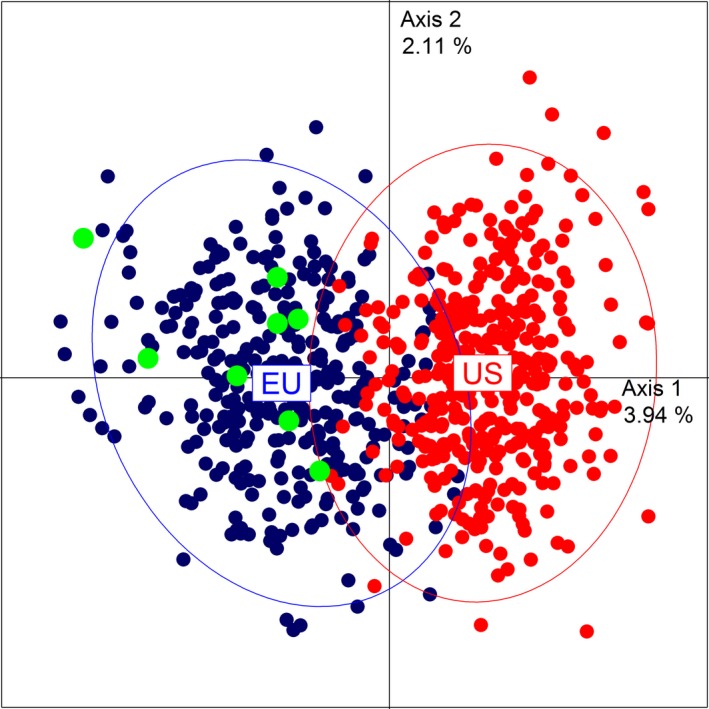
Principal component analysis performed at the individual level. European individuals were plotted using blue dots, whereas American individuals were plotted with red and green dots. Green dots represented American individuals located within the European dot cloud beyond the limit of the ellipse related to American individuals. Ellipses were plotted to illustrate the identified genetic clusters. They encompassed roughly 95% of the individuals of each range

First we used a PCA (Figure 1) to analyze the position of individuals on the factorial plan. Axis 1 roughly separated European (in blue) and American (in red and green) individuals in two partially overlapping clusters. A few American individuals (in green) were present inside the European dot cloud beyond the limit of the ellipse, whereas the contrary is not true. These American individuals belonged to four American populations (Altoona PA, Eriline KY, US Grp 3 VA, Wayne National Forest OH) located in the Northern Appalachian part of the native distribution.

**Figure 2:**
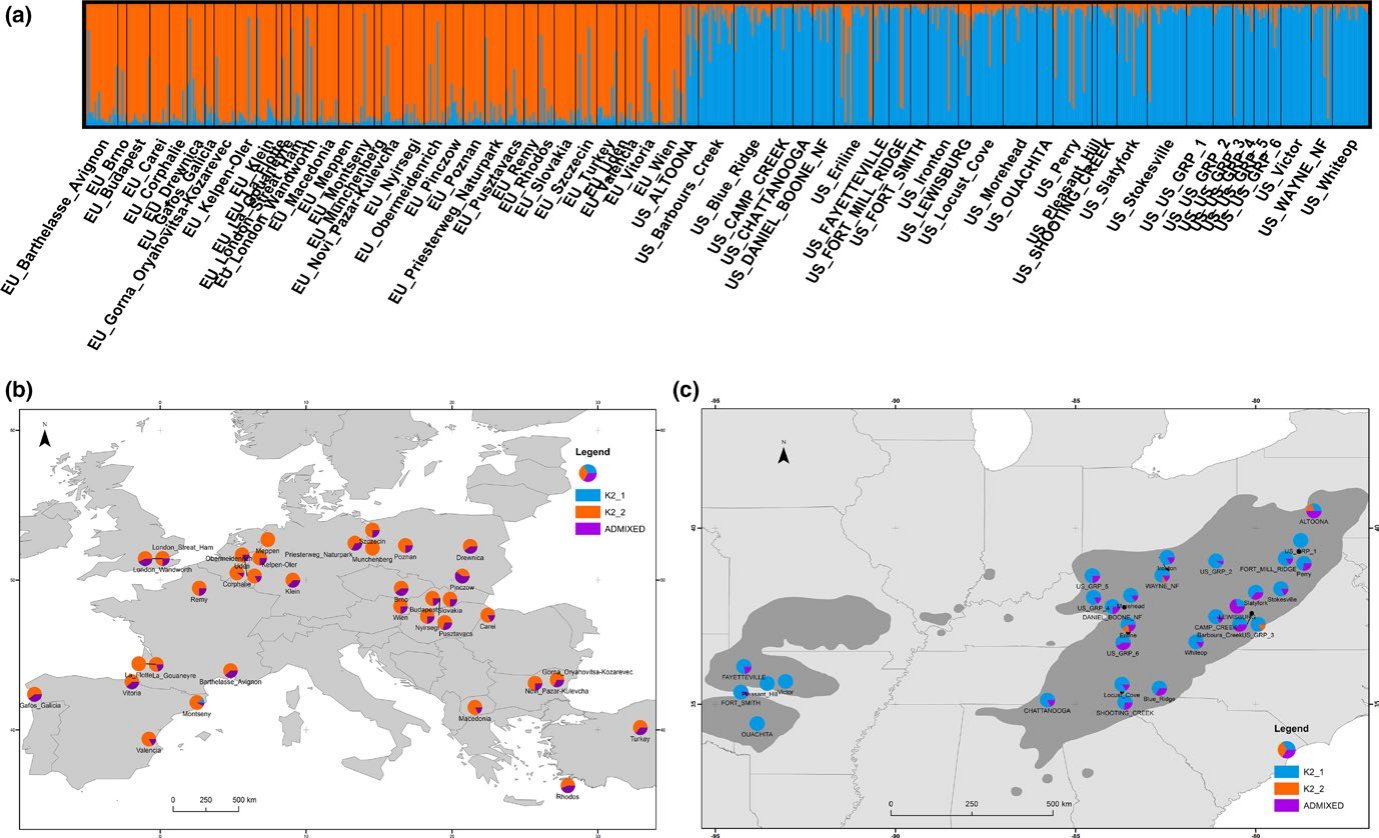
(a) Individual assignation for the most likely number of clusters (where *K* = 2) as a result of the between range STRUCTURE analysis. Each colored vertical line represents one individual ancestry membership between the two clusters (orange, cluster K2_1, and blue cluster K2_2). Black vertical lines separate different populations. Both analyses were computed on the initial dataset after clone removal (720 individuals from 63 populations genotyped using 113 SNPs). (b and c) Pie charts of the population assignation in Europe and the USA for the most likely number of clusters (where *K* = 2) as a result of the STRUCTURE analysis between ranges. In blue, proportion of individuals significantly assigned to cluster K2_1; in orange, proportion of individuals significantly assigned to cluster K2_2; and in Purple, proportion of individuals admixed in each population. The native distribution of black locust within America (Little, 1971) is plotted in gray shading and in Europe it is present almost everywhere from Southern to Northern Europe

Second, we used STRUCTURE to cluster the individuals according to molecular genetic similarity. An optimal number of *K* = 2 clusters (Supporting Information Appendix S2A,B) was identified in STRUCTURE output using the initial dataset after clone removal (720 individuals from the 63 populations from both ranges). In the first cluster (Figure 2, in blue; K2_1; Supporting Information Appendix S3), the majority of individuals came from America (76.8%), and only one European population (Montseny: 8.3%) contained samples that could be significantly attributed to this cluster using the individuals’ ancestry confidence intervals (0.245% of the total number of European individuals).

The second cluster (Figure 2c in orange; K2_2; Supporting Information Appendix S3) contained most of the European individuals (73.6% of the total number of European samples) and a few samples from North America (2.18%). The American samples that could be significantly assigned to cluster K2_2 belonged to only four American populations (Supporting Information Appendix S3), and only a small proportion of individuals were significantly attributed to cluster K2_2 (Altoona PA: 20%, Eriline KY: 18.2%, US Grp 3 VA: 16.7%, Wayne National Forest OH: 8.33%). These four populations were all located in the northern part of the Appalachian Mountains (Figure 2c). Finally, admixed individuals (i.e., those not attributed significantly to one cluster) were found in both ranges with relatively similar proportion (Europe: 26.1%, America: 21.0%, Global: 23.8%). In America, the proportion of admixed individuals per population ranged from 0% (Ouachita, Pleasant Hill, US Grp 1, US Grp 3, Victor) to 71% (Lewisburg), whereas in Europe it ranged from 0% (Meppen, Munchenberg) to 60% (Pinczow).

### 3.3 Significant genetic differentiation among populations in the native range, contrary to the introduced range

There was a significant genetic differentiation among all populations: Estimated *F*_ST_ among all populations was 5.23% (95% CI: 4.77%–5.70%). Overall, within the native range, black locust populations were clearly genetically differentiated, matching with geographic structure, whereas in the introduced European range, the differentiation between populations was low and no structure was detected across the continent.

**Figure 3:**
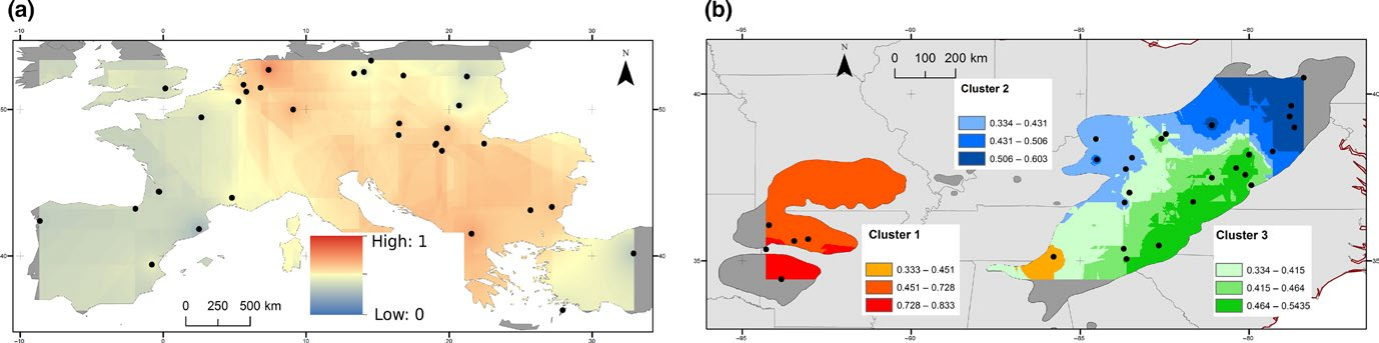
(a and b) The graphical IDW interpolation computed on the STRUCTURE for individual ancestry membership for each within range analysis. Results are shown for the most likely *K* in Europe and in the USA, *K* = 2 and *K* = 3, respectively. IDW within America is plotted over native distribution of black locust. The two European clusters are represented by a continuous color scale from blue (K2_1_EU) to red (K2_2_Eu). The three clusters in the USA are represented by a continuous red color scale (K3_1_US) through to a blue color scale (K3_2_US) and green color scale (K3_3_US)

Genetic differentiation among American populations was significant with an estimated *F*_ST_ of 4.46% (95% CI: 3.94%–5.06%). Clear signals of isolation by distance (IBD) were observed between populations in the native range, as both correlation tests were significant (Table 2; Supporting Information Appendix S4A). IBD remained significant among the Appalachian populations, but not among the Ozark populations. In the native range, the STRUCTURE analysis indicated that populations formed an optimal number of *K* = 3 clusters (Supporting Information Appendix S2B). The first cluster (K3_1_US, Figure 3b, Supporting Information Appendix S5) corresponded to the Ozark western populations with high membership coefficients at the population level; the individuals from Ozark populations were mostly assigned to this cluster, with a minimum number of individuals for Fayetteville (22.2% of individuals assigned to K3_1_US and 77.8% of admixed individuals) and a maximum for Fort Smith (80.0% of individuals assigned to K3_1_US and 20% of admixed individuals). The mean admixture among populations assigned to this cluster was 51.2%.

**Table 2:** Isolation by distance correlation tests

Range	Pearson test		Mantel test	
	*r*	*p*	*r*	*p*
USA	**0.53**	**2.9 × 10^−30^**	**0.479**	**3 × 10^−4^**
Appalachians	**0.193**	**1.28 × 10^−3^**		
Ozarks	0.386	0.27		
Europe	*r*	0.75	−0.028	0.562
K2_1:	0.105	0.223		
K2_2:	0.0692	0.483		

Note

Both regression of pairwise *F*_ST_/(1 − *F*_ST_) on logarithm of pairwise geographic distance (Rousset, 1997) and a Mantel test were performed within each range or within a subselection of the population in each range. Significant results are in bold.

The second cluster (Figure 3b, K3_2_US, Supporting Information Appendix S5) was located in the north of the Appalachian Mountains over Kentucky, Maryland, Ohio, Pennsylvania, Virginia, and West Virginia. The individuals assigned to this cluster were mainly found in North Appalachian populations (Altoona, Perry, US Grp 3 and US Grp 4) and ancestry membership gradually decreased toward the south and the east of the Appalachian Mountains. A proportion of individuals within the populations assigned to this cluster were significantly assigned, ranging from 4.5% (Stokesville) to 50% (Us Grp 4). Four populations also partially comprised individuals assigned to other clusters: 6.7% of US Grp 1 individuals and 16.7% of US Grp 4 individuals were assigned to cluster K3_1_US, and 5.6% of Locust Cove individuals were assigned to the cluster K3_3_US. Mean admixture among populations assigned to this second cluster was 77.5%.

The third cluster (Figure 1c, K3_3_US) was mainly formed in the eastern part of the Appalachians Mountains in North Carolina (Blue Ridge), Virginia (Barbours Creek, Whiteop), and in the southern part of West Virginia (Slatyfork). The other populations all comprised a fraction of individuals significantly assigned to this cluster with values ranging from 4.5% (Eriline) to 30% (Barbours Creek). The mean admixture among populations assigned to this cluster—with the exception of the 2 fully admixed populations—was 83.9%.

Genetic differentiation among European populations was significant with an estimated *F*_ST_ of 3.08% (95% CI: 2.60%–3.60%), which was significantly lower than the *F*_ST_ observed in the native range. No significant signal of IBD was found either by testing the correlation between pairwise *F*_ST_/(1−*F*_ST_) ratio and the logarithm of pairwise geographic distances (Supporting Information Appendix S4B; Pearson correlation test, *r* = −0.013 *p* = 0.75) or by realizing a Mantel test (Table 2; *r* = −0.028, *p* = 0.562). The correlation was not significant within each cluster either (Table 2; K2_1: Pearson correlation test, *r* = 0.105, *p* = 0.223, K2_2: Pearson correlation test, *r* = 0.0692, *p* = 0.483).

In the introduced range, the STRUCTURE analysis indicated that populations formed an optimal number of *K* = 2 clusters (Supporting Information Appendix S2C).

The first cluster (Figure 3a in red; K2_1_EU Supporting Information Appendix S5) was composed of 14 populations mainly from Central Europe. All populations in this cluster contained a fraction of significantly assigned individuals with values ranging from 8.33% (Gorna) to 62.5% (Meppen). Moreover, two populations (Macedonia, Nyirsegi) had 8.33% of their individuals significantly attributed to the second European cluster (K2_2_EU). Overall, the mean individual admixture in populations assigned to this cluster, with the exception of the fully admixed population, was 75.6%, to which 19% of individuals were significantly assigned, while 5.3% were assigned to the other cluster.

The second cluster (Figure 3a in blue; K2_2_EU Supporting Information Appendix S5) was mainly represented by 15 populations from Western and Eastern Europe. All populations contained a fraction of individuals which could be significantly assigned to this cluster, with values ranging from 9.1% (Turkey) to 100% (Munchenberg). Moreover, four populations had some individuals significantly attributed to the first European cluster (K2_1_EU): Vitoria (7.7%), Wien and Szczecin (8.33%), and Valencia (16.7%). Overall, mean individual admixture among populations assigned to this cluster was 70.9%.

### 3.4 MAF distribution and detection of a bottleneck in the introduced populations

The MAF analysis performed on the initial and additional datasets highlighted a deficit in low frequency alleles when using 113 SNPs (MAF mode: 0.05–0.15) in both the native and invasive ranges, confirming that with this initial set we had oversampled high MAF SNPs, biasing the evaluation of diversity indexes (Supporting Information Appendix S6A). Consequently, the number of SNPs was increased to 251 SNPs by genotyping low MAF SNPs from our initial set of SNPs (Verdu et al., 2016) on a reduced number of samples.

When analyzing the 251 SNPs in 163 samples, no difference in heterozygosity was observed between either range. However, a lower AR was observed in Europe compared to North America (1.91 vs. 1.96, Wilcoxon, v = 1,375.5, *p* = 0.00453), as well as a lower total number of alleles in Europe compared to North America (485 vs. 494; Mann–Whitney, W = 39,802, *p* < 0.0001) (Supporting Information Appendix S6B).

## 4 DISCUSSION

Due to an extensive population genetics analysis in both native and European invasive ranges, we were able to show that black locust was likely to have been introduced to Europe from a limited part of its northeastern native distribution in the Appalachian Mountains. This founding effect brought about a bottleneck, detected only when we increased the number of SNPs with low MAF markers. A strong genetic structure was observed in the USA, whereas a much weaker one was detected in Europe. Moreover, asexual propagation was probably more prevalent in the invasive range than in the native one.

### 4.1 Populations genetics and introduction history: European black locust populations close to Northern Appalachian populations

The genetic results suggest that the black locust was introduced to Europe from a restricted number of native populations located in the northeastern Appalachian Mountains. The robustness of this result is due to an intensive sampling effort on three levels: number of populations (29 in Europe and 34 in USA), number of individuals (334 in Europe and 386 in USA without clones, with a mean of 11.4 samples per population), and number of markers (113 SNPs). Furthermore, historical reviews and two types of analyses (PCA, Structure) led to the same conclusion concerning the limited origin of European populations.

The results obtained using a molecular approach are congruent with historical records pointing to the original sources of black locust in the northeastern part of its native range in the Appalachian Mountains. By reviewing historical studies, we were able to conclude that the first black locusts introduced to Europe during the early 17th century were likely to have come from Virginia and further black locusts introduced during the 17th and 18th centuries from Pennsylvania and West Virginia (Cobbett, 1825; Gunther, 1922; Michaux, 1813). Consequently, taking into account both the historical indications and the genetic proximity of all European black locust populations to a few native ones, it can be hypothesized that no subsequent introductions followed, and that the expansion of the species in Europe through asexual reproduction or seeds resulted from the original black locusts grown in Europe.

### 4.2 Evidencing the bottleneck depends on the set of genotyped SNPs

Given that there are a few American populations close to European ones, a bottleneck is expected in European populations. The decrease in genetic diversity was only observed when using a larger number of SNPs, due to some particular properties of SNPs.

SSRs and SNPs are two widely used markers for genotyping non‐model species (Morin, Luikart, & Wayne 2004; Coates et al., 2009; Helyar et al., 2011). SNPs have many advantages: They can be easily developed using NGS; genotyping is easily replicable among laboratories; and SNPs are widely distributed throughout the genome (Coates et al., 2009; Morin et al., 2004; Helyar et al., 2011). However, more SNPs are needed than SSRs in order to reach the same level of precision, essentially because SSRs are multiallelic, whereas SNPs are mainly biallelic (Morin et al., 2004). One major problem in using SNPs is the ascertainment bias (Coates et al., 2009; Morin et al., 2004; Helyar et al., 2011). In particular, SNPs with a high minor allele frequency are more susceptible to being sampled for genotyping populations and consequently this can alter diversity estimates (Helyar et al., 2011). As a consequence, a genetic diversity analysis conducted with SNP data may lead to false negative or false positive conclusions (Helyar et al., 2011; Morin et al., 2004). Some empirical studies showed that SNPs performed better than SSRs for studying population structure, whereas SSRs were more efficient for estimating diversity (Singh et al., 2013). Nevertheless, other empirical studies reached the same conclusion regardless of type of marker used (van Inghelandt, Melchinger, Lebreton, & Stich 2010; Filippi et al., 2015).

Our study emphasizes the importance of taking the SNP ascertainment bias into account when comparing genetic diversity among several groups. Using the initial dataset, we observed more frequent minor alleles (modal class 0.05–015, Online Resource 3), which confirmed the sampling bias. We partially corrected this bias by using the additional dataset (251 SNPs), where actual MAF distribution is closer to the expected MAF distribution (modal class 0–0.05, Supporting Information Appendix S6A). By carrying out the analysis with this additional dataset, we were able to detect a bottleneck (decrease in allelic richness and total number of alleles in the introduced range, Supporting Information Appendix S6B), which would not have been the case if we had only used the initial dataset for studying genetic diversity between ranges (no difference in heterozygosity, allelic richness, and total number of alleles between ranges, Supporting Information Appendix S6B). Both a loss in allelic richness and in the total number of alleles point to the occurrence of a bottleneck, whereas heterozygosity is not expected to respond as well as allelic richness to a founding event (Dlugosch et al., 2015) as observed in our study.

Uller and Leimu (2011) demonstrated that genetic variation between native and invasive ranges was influenced by taxonomy: Invasive animals often suffered a loss of genetic diversity between the ranges, whereas invasive plants often exhibited higher genetic diversity in the invasive range (Uller & Leimu, 2011). According to these authors, one factor contributing to this pattern is that invasive animal populations are often founded by single introduction events, whereas multiple introductions associated with admixture are more common for plants (Uller & Leimu, 2011). Therefore, our results show that, similar to plants, black locust follows the original pattern of an introduction from a few populations and a loss of genetic diversity. Many studies investigating the origins of herbaceous invasive plants have documented multiple introductions from wide areas of their native range. For the European weed *Ambrosia artemisiifolia*, multiple introductions from two distinct genetic clusters of the native American range have been evidenced (van Boheemen et al., 2017). Similarly, two clusters in Western and Eastern Europe have been identified as the sources of the American weed *Centaurea solstitialis* (Barker, Andonian, Swope, Luster, & Dlugosch, 2017). Thirdly, a wide scale study of the invasive weed *Mikania micrantha *in South‐East Asia demonstrated the existence of two distinct genetics clusters that resulted from separate introductions originating from the native American range (Yang et al., 2017). Studies on invasive trees are less numerous, but they have generally concluded that multiple introductions occurred (Besnard et al., 2014; Merceron et al., 2017; Pairon et al., 2010; Thompson et al., 2015) without being able to clearly identify the population sources. A recent study on the genetic structure of invasive populations of *Acacia saligna* within several invasive ranges suggested that multiple introductions occurred from populations distributed throughout the Australian native range (Thompson et al., 2015).

### 4.3 Structure in the native ranges was shaped by long‐term evolutionary processes, whereas structure in the invasive range reflects anthropic action

Natural evolutionary processes seemed to have shaped the genetic diversity and structure of black locust populations in the native range.

Three genetic clusters were identified within the native range, with the greatest differentiation between the first cluster in the Ozark Mountains and the two clusters in the Northern and Southern Appalachian Mountains. Together with the pattern of isolation by distance, this suggests the action of natural and long‐term evolutionary processes. On the contrary, in Europe, the weak structure in two clusters with a few outlying populations and no isolation by distance would suggest a recent evolutionary history marked by human actions.

The genetic structure detected in the American black locust is congruent with observations in other North American tree species. In North America, glacial refugia have been identified on both sides of the Mississippi River (Hewitt, 2000; Swenson & Howard, 2005) throughout geographic areas closely related to the genetic clusters identified in this study. A large differentiation on each side of the Mississippi River has been recorded for at least one other tree species, the loblolly pine (*Pinus taeda* L.), which exhibited two distinct genetic clusters (Lu et al., 2016). It is likely that the Mississippi River acted as a physical barrier during postglaciation recolonization after the last glacial maximum during Wisconsinan 21,000 years ago (Pessino, Chabot, Giordano, & DeWalt, 2014).

Moreover, similar to our findings, distinct genetic clusters have been identified along a north–south axis of the Appalachian mountains in several woody species, such as *Scirpus ancistrochaetus* (Cipollini, Lavretsky, Cipollini, & Peters, 2017), *Tsuga caroliniana* (Potter, Campbell, Josserand, Nelson, & Jetton, 2017) and *Pinus strobus* (Nadeau et al., 2015). As described by Swenson and Howard (2005), a historical suture zone has been identified between the Northern Appalachian Mountains and Southern Appalachian Mountains. Consistent with this suture zone, two glacial refugia were detected for *P. strobus* in the Northern and Southern Appalachian Mountains. (Nadeau et al., 2015). It can therefore be assumed that Appalachian black locust genetic structure was driven by the same processes as for other North American trees and reflects postglacial colonization routes originating from glacial refugia on each side of the Appalachian Mountains.

On the contrary, in Europe the weak structure in two clusters with a few outlying populations and no isolation by distance would suggest a recent evolutionary history marked by human actions.

Within the European range, two distinct genetic clusters were detected throughout Central Europe (Austria, Bulgaria, Czech Republic, Central Germany, Hungary, Macedonia, the Netherlands, Slovakia, and Romania) and in Western and Eastern Europe (England, France, Eastern Germany, Poland, Spain and Turkey), but with a very weak signal (within Europe *F*_ST_ = 3.03%). Most of the individuals were admixed between these two clusters (approx. 75%) and within each cluster one outlying population drove the signal of differentiation: Meppen (62.5% of individuals assigned to K2_1) and Munchenberg (100% of individuals assigned to K2_2). Although weak, three signals confirmed that the observed genetic structure was significant: (a) Within each cluster, some individuals were significantly assigned to the cluster, (b) the STRUCTURE admixture parameter (*α*) was checked for convergence (*α* should be relatively constant with a range of 0.2 or less −)as recommended in the STRUCTURE manual (Pritchard, 2010), and (c) when the two outlying populations were removed, similar genetic structure results were observed within Europe (data not shown).

As previously discussed, the black locust was most probably introduced to Europe from only a few populations located in a limited area of its native range in the northern part of the Appalachian Mountains. No support was found for the hypothesis that an introduction from two different American genetic clusters could have founded the two European clusters. Historical writings indicate that, in the 17th century, Vespasien Robin disseminated seeds collected from black locust trees grown in Paris throughout Europe, and that as from 1,634, the black locust was planted all over Europe for ornamental purposes. Seed orchards were created in Europe by trading European raised seeds (Cierjacks et al., 2013, Cobbett, 1825; François de Neufchateau, 1807) thus propagating the same genetic material throughout Europe. This can lead us to conclude that the origins of the central European cluster are related to human selection. As from the second half of the 18th century, extensive afforestation programs have been conducted in central Europe (in particular Germany, Hungary, and the Czech republic) promoting black locust for forestry purposes (Cierjacks et al., 2013; Vítková, Müllerová, Sádlo, Pergl, & Pyšek, 2017). Moreover, a genetic breeding program has been conducted since the beginning of the 20th century in Hungary (Keresztesi, 1983). The genetic clustering within Europe may result from the evolution caused by artificial selection due to human‐oriented selection and tree breeding, which was initiated in Central Europe in the 18th century. Thus, we can say that the European black locust is partially domesticated, and we can ask which traits influenced invasiveness. Further investigations involving common garden surveys would be necessary in order to assess whether genetic differences resulted in phenotypic differences.

### 4.4 The role of clonality in shaping genetic diversity in Europe

In general, we found that European populations of black locust were more clonal than American populations. This was demonstrated by comparing the number of clones detected within each range, as well as the higher index of clonal diversity R, and the analysis of the inbreeding coefficient *F*_IS_. The same sampling protocol was followed by all field workers, who respected a minimum distance of 25 m between sampled individuals; it can therefore be concluded that the black locust is able to spread more than 25 m by clonal propagation. This result was confirmed when visualizing the mapping of individuals with GPS coordinates (data not shown, available upon request). A lower *F*_IS_ in European populations than in American populations indicated an excess of heterozygosity within the former. Clonality usually produces this pattern (Arnaud‐Haond et al., 2007; Halkett, Simon, & Balloux, 2005; Stoeckel & Masson, 2014), as clonal reproduction can maintain heterozygosity over generations (Stoeckel et al., 2006). However, no significant relationship between the clonal diversity index (i.e., R) and *F*_IS_ was evidenced (data not shown).

Moreover, within the European range, the two outlying populations, Meppen and Munchenberg, were clearly differentiated no matter which of the analyses was applied. The results in a previous study on the Munchenberg population (code N° 7 – Hasenholz in Liesebach et al., 2004) showed that this population could clearly be differentiated from all the others. In addition, two major clones were detected which covered 80% of the stand (Liesebach et al., 2004). This is consistent with our finding since the Munchenberg population exhibited the lowest negative *F*_IS_ (−0.180), which indicated an excess of heterozygosity potentially due to a high level of clonality.

In Japan, both clonal and sexual reproduction have been found to promote the spread and invasion of black locust (Kurokochi & Hogetsu, 2014). Sexual regime is likely to influence invasiveness and a shift in the mating system has already been observed between ranges for several invasive species (Barrett, Colautti, & Eckert, 2008; Petanidou et al., 2012; Rambuda & Johnson, 2004). Clonal populations can maintain a high level of genetic diversity; however, they can be sensitive to founder events (Barrett, 2010). Clonality is likely to strongly decrease *F*_ST_ and to slowly decrease genotypic diversity in purely clonal populations (Balloux, Lehmann, & De Meeûs, 2003), but partially clonal populations are hard to differentiate from strictly sexual populations (Balloux et al., 2003). A theoretical study showed an advantage of clonal reproduction for species invasiveness. However, the relationship is not linear and species combining a high clonal rate with a small rate of sexual reproduction would have a higher invasiveness (Bazin, Mathé‐Hubert, Facon, Carlier, & Ravigné, 2013). Clonal reproduction provides invasive plant species with reproductive assurance (Barrett et al., 2008). Shifts in the mating system from outcrossing to clonality, due to a strong founder event, have been observed for the invasive *Eichhornia crassipes* (Barrett et al., 2008) and *Fallopia japonica* (Hollingsworth & Bailey, 2000). However, this is not systematic and some pure outcrossing species are successful invaders, such as *A. artemisiifolia* (Friedman & Barrett, 2008). It is also possible that a shift toward more clonal reproduction occurred in the mating system of the black locust between the native and the invasive range. This could have been produced by the founder event or by artificial selection, as one traditional way of managing black locust plantations in Europe was to stimulate clonal reproduction by damaging tree roots (François de Neufchateau, 1807; Saint‐Jean de Crève & Coeur, 1786).

## 5 CONCLUSION

We found a remarkable congruence between our genetic analysis and historical records regarding the geographic origins of the European black locust, with both approaches pointing to European populations originating from the Northern Appalachian Mountains. The history of black locust introduction is thus a unique pattern among invasive trees, which are commonly characterized by multiple introduction events. As a consequence, only a small part of black locust genetic diversity was introduced to Europe from its native American range.

Furthermore, in spite of the fact that black locust suffered a genetic bottleneck and a loss of diversity following introduction, this did not prevent its successful colonization throughout its European range. Moreover, we found some evidence for a shift in mating systems between ranges with an increase in clonality in Europe, resulting from either natural or artificial selection. However, our sampling method was not specifically designed to investigate changes in clonality during and after introduction. Therefore, further studies involving the sampling of extensive populations and plots would be needed to better understand the role of clonality in the success of this species, conjointly with studies on the role of sexual reproduction in the spread of the black locust.

## CONFLICT OF INTEREST

None declared.

## AUTHOR CONTRIBUTION

AM, AJP, LL and SM conceived the experiment. XB, CFV, PB, CM, AM, MVL and SM sampled the populations. XB, CFV, AD, EG, MP and SM made the lab work. XB analyzed the data, with the contribution of CFV, EA, KD, ODT and SM. XB wrote the paper, with the contribution of AJP and SM. All authors read and approved the submitted version. XB and SM are co‐corresponding authors.

## Data Availability

Data are accessible on Open Science Framework repository https://osf.io/k97ax/ and they are publicly available.
