# Supplementary material for: A few north Appalachian populations are the source of European black locust
Source: Ecol Evol. 2019 Feb 16;9(5):2398–414. doi: 10.1002/ece3.4776 (PMC6405530; doi:10.1002/ece3.4776)

A – ΔK plot produced by STRUCTURE HARVESTER for the 10 runs from K = 1 to 20 of the whole data set (US + European popualtions. Most likely K is for K = 2.


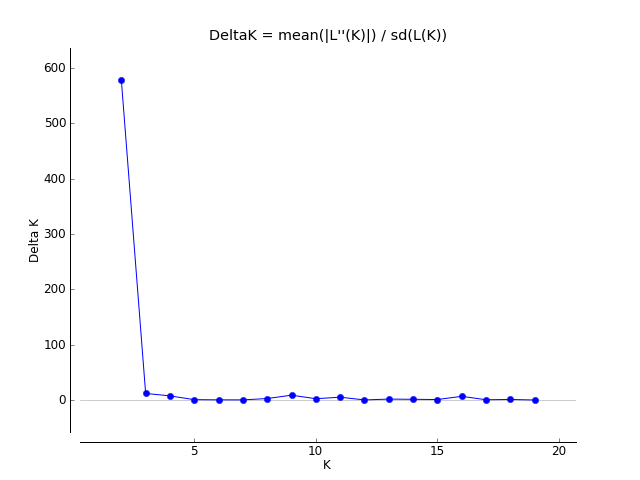


B – ΔK plot produced by STRUCTURE HARVESTER for the 10 runs from K = 1 to 20 of the US data (US populations). Most likely K is for K = 3.


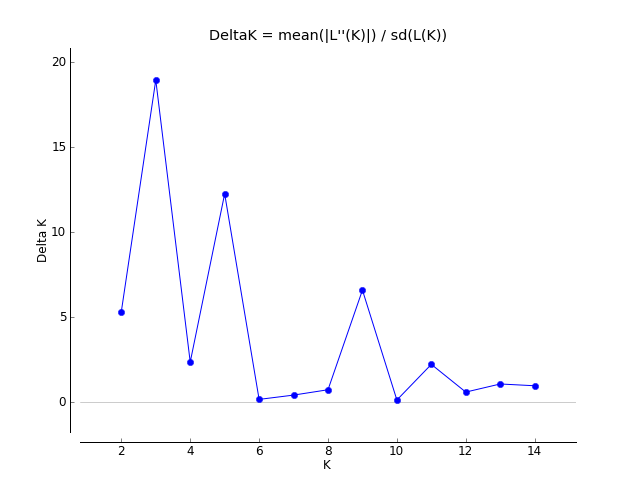


C – ΔK plot produced by STRUCTURE HARVESTER for the 10 runs from K = 1 to 20 of the European data (European populations). Most likely K is for K = 2.


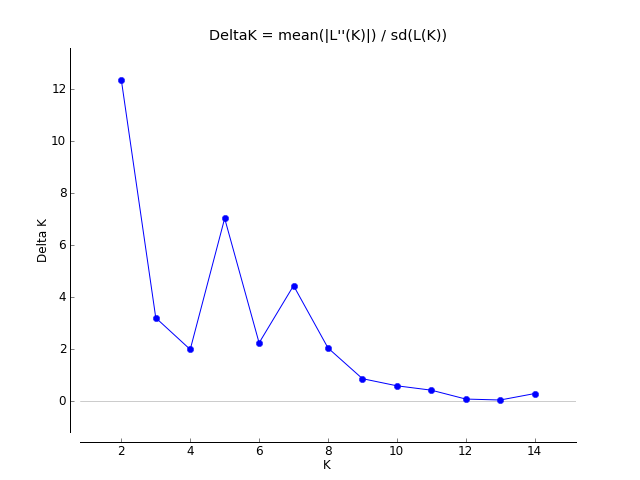

Supplement: Supplementary file 2 [file ECE3-9-2398-s002.docx]
