# Supplementary figures and images for: A few north Appalachian populations are the source of European black locust
Source: Ecol Evol. 2019 Feb 16;9(5):2398–414. doi: 10.1002/ece3.4776 (PMC6405530; doi:10.1002/ece3.4776)

A – Isolation By Distance in America.


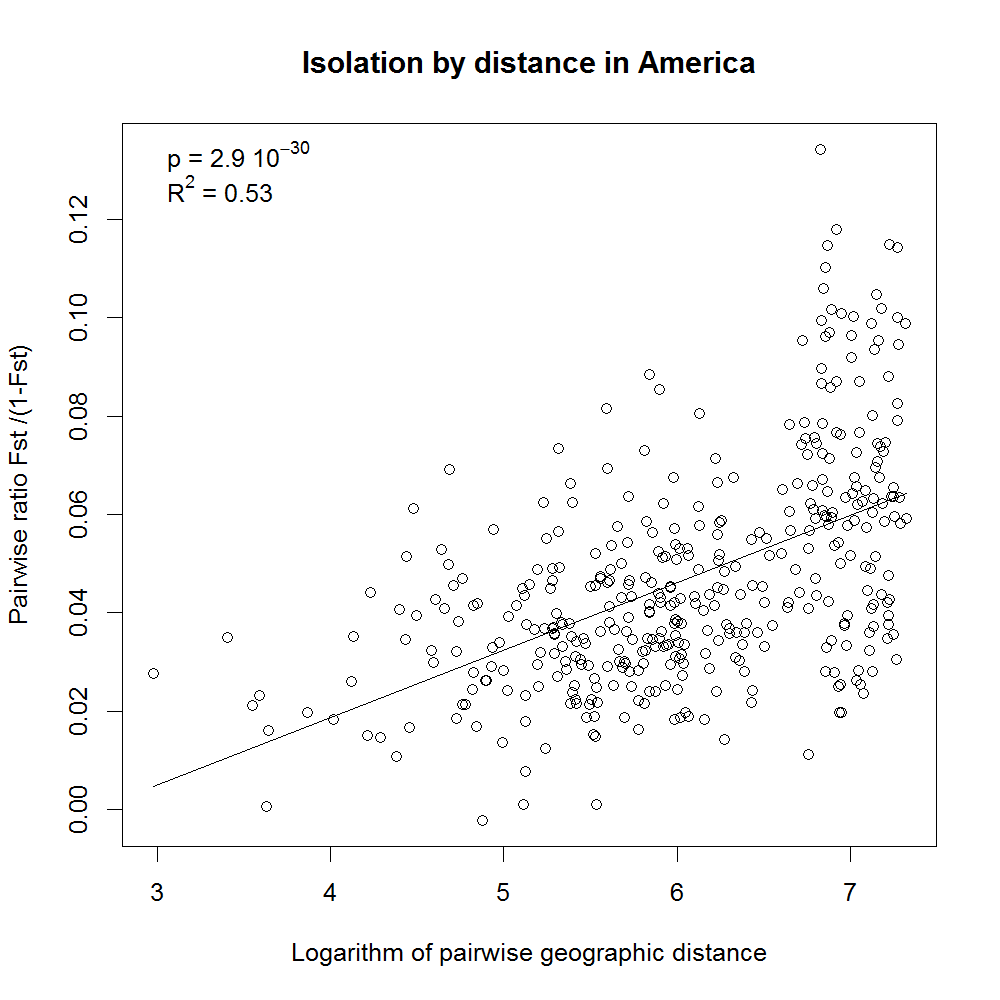


B – Isolation By Distance in Europe
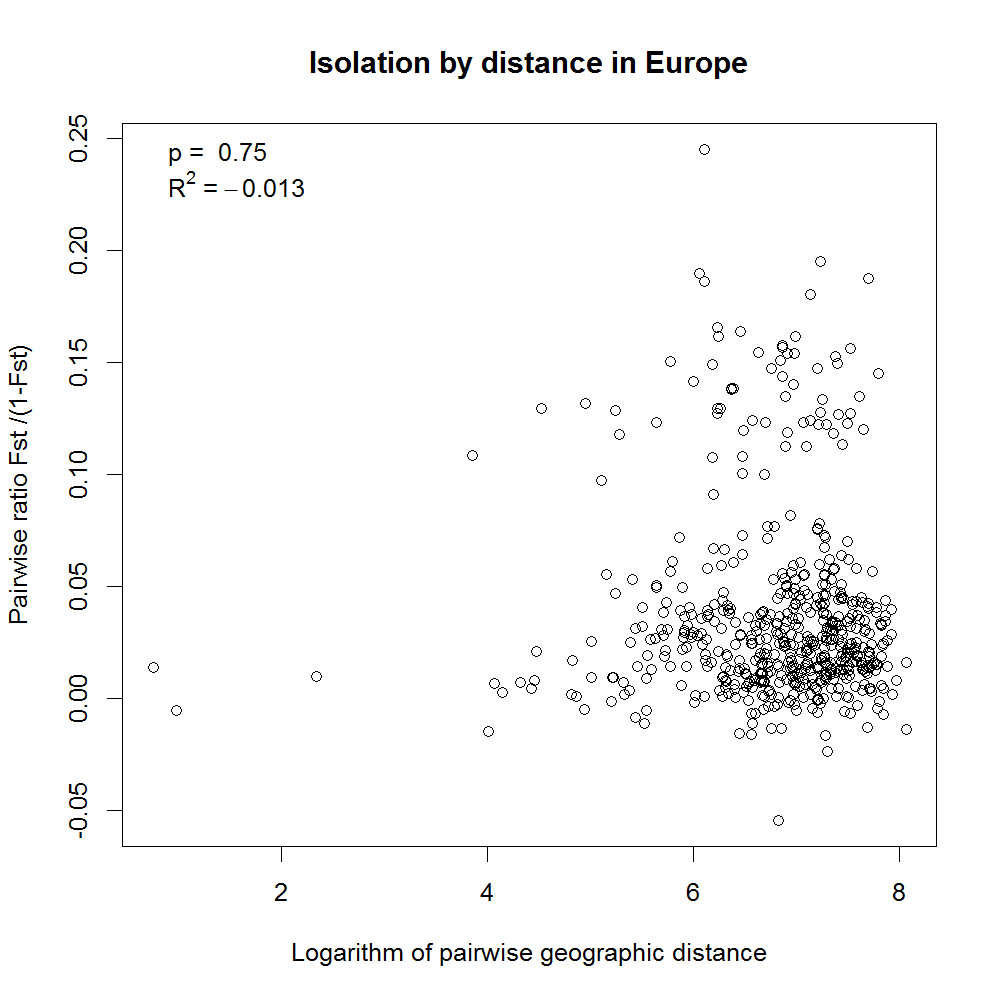

Supplement: Supplementary file 4 [file ECE3-9-2398-s004.docx]
