# Supplementary material for: A few north Appalachian populations are the source of European black locust
Source: Ecol Evol. 2019 Feb 16;9(5):2398–414. doi: 10.1002/ece3.4776 (PMC6405530; doi:10.1002/ece3.4776)
Supplement: Supplementary file 6 [file ECE3-9-2398-s006.docx]

A)

|  | European range | American range | Both ranges |
| --- | --- | --- | --- |
|  | A | B | C |
| Initial dataset | 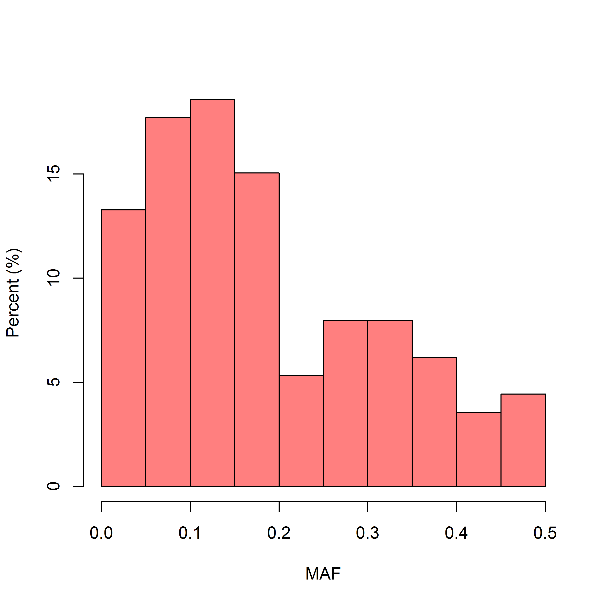 | 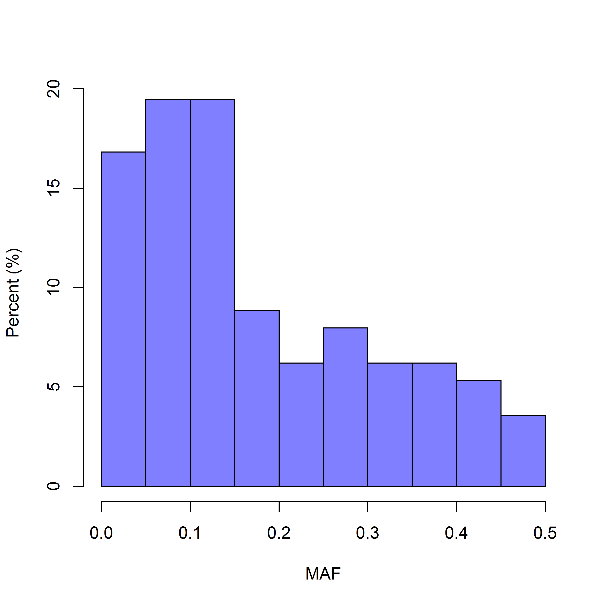 | 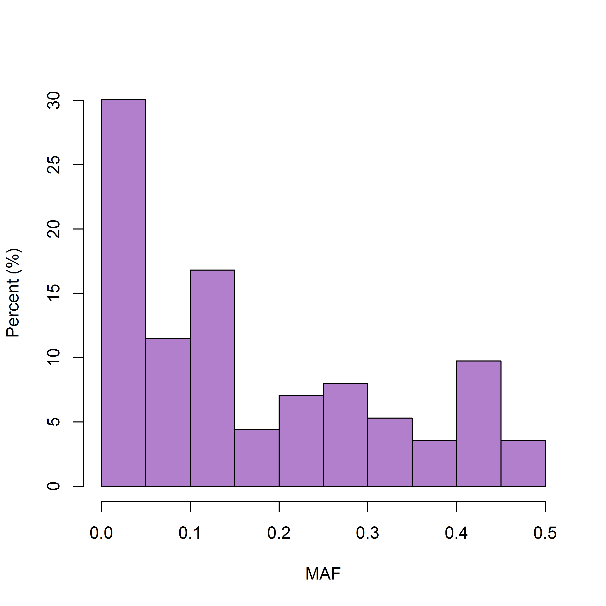 |
|  | D | E | F |
| Additional dataset | 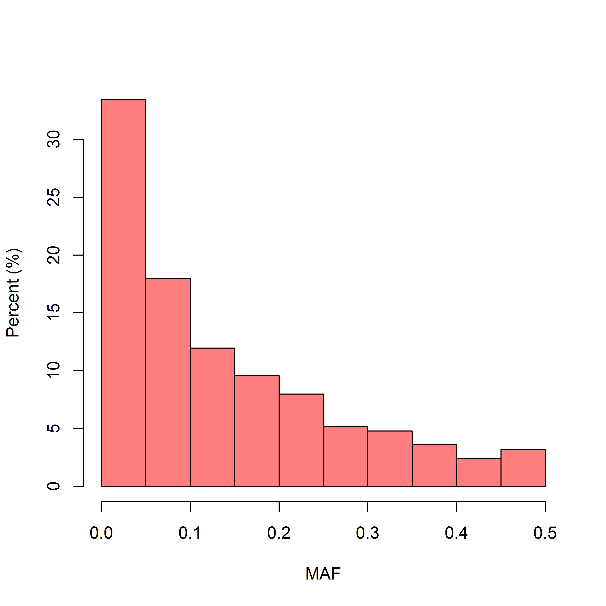 | 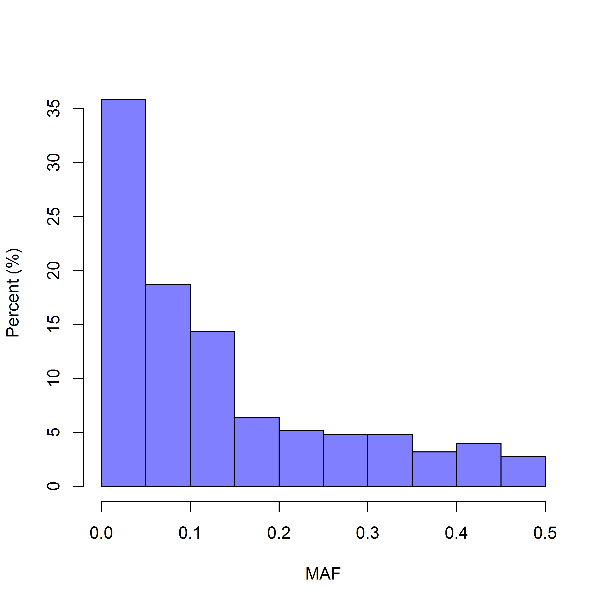 | 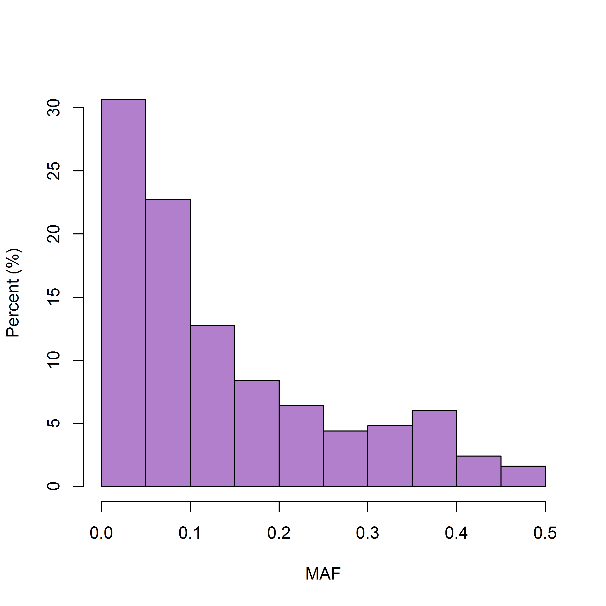 |

B)

|  | Range | Ho | He | TNA | AR |
| --- | --- | --- | --- | --- | --- |
| Initial Dataset  (113 SNPs) | EU | 0.24 | 0.27 | 226 | 2.00 |
|  | US | 0.22 | 0.26 | 226 | 2.00 |
| Additional Dataset (251 SNPs) | EU | 0.19 | 0.21 | **485** | **1.91** |
|  | US | 0.17 | 0.20 | **494** | **1.96** |
