# Supplementary material for: A few north Appalachian populations are the source of European black locust
Source: Ecol Evol. 2019 Feb 16;9(5):2398–414. doi: 10.1002/ece3.4776 (PMC6405530; doi:10.1002/ece3.4776)
Supplement: Supplementary file 7 [file ECE3-9-2398-s007.docx]

Supplementary information

1. A) Complete information about the sampled populations. Range corresponds either to Europe (EU) or the USA (US) and either the country or the state is indicated. N is the number of individuals genotyped per population. GPS coordinates were provided using the WGS84 geographic projection. Altitude was extracted from the Worldclim database (Fick & Hijmans, 2017) using the geographic coordinates. Information regarding harvesting is as follows: harvesting date, harvester names, funding source, stand type (natural or Common Garden) and supplementary details regarding the forest location. B) Populations are defined using the trees sampled in the Michigan State University common garden. X Long. Used and Y Lat. Used refer to the geographic coordinates of the created population (population name) which were obtained using the WGS84 geographic projection. “Original” columns refer to the original sampling location and coordinates of the seeds. Trees from close original geographic location were gathered within the same created population in order to obtain a sufficient number of individuals within each population.
2. ΔK calculated according to the Evanno et al., (2005) method on the STRUCTURE output using STRUCTURE HARVESTER (Earl & vonHoldt, 2012). A – L(K) plot produced by STRUCTURE HARVESTER for the 10 runs from K = 1 to 20 of the whole dataset (US + European populations). Large increase in the L(K) between 1 and 2 indicates that K = 2 is a pertinent number of clusters for synthetizing the dataset. B – ΔK plot produced by STRUCTURE HARVESTER for the 10 runs from K = 1 to 20 of the whole dataset (US + European populations). Most likely K is K = 2. C – L(K) plot produced by STRUCTURE HARVESTER for the 10 runs from K = 1 to 20 of the US data (US populations). D – ΔK plot produced by STRUCTURE HARVESTER for the 10 runs from K = 1 to 20 of the US data (US populations). Most likely K is K = 3. E – L(K) plot produced by STRUCTURE HARVESTER for the 10 runs from K = 1 to 20 of the European data (European populations). C F – ΔK plot produced by STRUCTURE HARVESTER for the 10 runs from K = 1 to 20 of the European data (European populations). Most likely K is K = 2.
3. Proportion of individuals in each population assigned to the K clusters of the most likely K, using the STRUCTURE analysis conducted on the within range datasets. K2_1: Proportion of individuals significantly assigned to cluster K2_1; K2_2: Proportion of individuals assigned to cluster K2_2; Admixed: Proportion of admixed individuals and Assignation_cluster: assignation cluster of the population according to the highest proportion of individuals significantly assigned.
4. IBD within America (A) and within Europe (B). ratio FST /(1-FST) from pairwise FST was calculated and plotted against the logarithm of the pairwise geographic distances among populations (Rousset 1997)
5. Proportion of individuals in each population assigned to the K clusters of the most likely K, based on the STRUCTURE analysis conducted using the within range datasets. Within America: K3_1_US: Proportion of individuals significantly assigned to cluster K3_1_US; K3_2_US: Proportion of individuals assigned to cluster K3_2_US; K3_3_US: Proportion of individuals assigned to cluster K3_3_US; Admixed_US: Proportion of admixed individuals and Assignation_cluster_US: assignation cluster of the population using the highest proportion of individuals significantly assigned. Within Europe: K2_1_EU: Proportion of individuals significantly assigned to cluster K2_1_EU; K2_2_EU: Proportion of individuals assigned to cluster K2_2_EU; Admixed_EU: Proportion of admixed individuals and Assignation_cluster_EU: assignation cluster of the population using the highest proportion of individuals significantly assigned.
6. A) MAF distributions assessed using the initial dataset after clone removal (720 genotyped individuals using 113 SNPs, A B and C) and using the additional dataset (163 genotyped individuals using 251 SNPs, D E and F). In blue for the American range, in red for the European range, in COLOR for both ranges together. G-Distribution of the Total number of alleles estimated by 1000 bootstrap replications over all loci and individuals; in blue the American range and in red the European range. B) Genetic diversity indices per range estimated using Fstat (Goudet 2013) and the additional dataset (163 genotyped individuals using 251 SNPs). Ho: Observed Heterozygosity, He: Expected Heterozygosity, AR: Allelic Richness, TNA: Total Number of Alleles. Bold characters indicate significant differences (p < 0.05).
